# Real-world outcomes in patients with tumor-induced osteomalacia treated vs not treated with burosumab

**DOI:** 10.1210/jendso/bvag165

**Published:** 2026-07-27

**Authors:** María Belén Zanchetta, Kathryn M Dahir, Erik A Imel, Thomas O Carpenter, Kerry Sandilands, Ben Johnson, Zhiyi Li, Lucinda Camidge, Elke Rottier, Emma Wilkes, Emily Vinall, Soraya Sader, Suzanne M Jan de Beur

**Affiliations:** Instituto de Investigaciones Metabólicas (IDIM), Universidad del Salvador, Buenos Aires C1012AAR, Argentina; Department of Endocrinology, Vanderbilt University Medical Center, Nashville, TN 37212, USA; Departments of Medicine and Pediatrics, Indiana University School of Medicine, Indianapolis, IN 46202, USA; Departments of Pediatric (Endocrinology), and Orthopedics and Rehabilitation, Yale University School of Medicine, New Haven, CT 06510, USA; Health Economics Outcomes Research, Kyowa Kirin International, Galashiels TD1 1QH, UK; Health Economics Outcomes Research, Kyowa Kirin International, Marlow SL7 1HZ, UK; HEOR, Rare Disease (Medical Affairs), Kyowa Kirin Inc., Princeton, NJ 08540, USA; Real World Evidence, Adelphi Real World, Bollington SK10 5JB, UK; Real World Evidence, Adelphi Real World, Bollington SK10 5JB, UK; Real World Evidence, Adelphi Real World, Bollington SK10 5JB, UK; Real World Evidence, Adelphi Real World, Bollington SK10 5JB, UK; Global HEOR, Ultragenyx Pharmaceutical Inc., Novato, CA 94949, USA; Division of Endocrinology and Metabolism, University of Virginia, Charlottesville, VA 22908, USA

**Keywords:** tumor-induced osteomalacia, burosumab, observational study, symptom burden, patient-reported outcome measures

## Abstract

**Context:**

Tumor-induced osteomalacia (TIO) is an ultra-rare condition caused by tumors secreting fibroblast growth factor-23, leading to chronic hypophosphatemia, pain, muscle weakness, fractures, and impaired physical function. Burosumab is a monoclonal antibody indicated for treatment of TIO when tumors cannot be curatively resected.

**Objective:**

This study compares clinical and patient-reported outcomes in patients with TIO treated vs not treated with burosumab in a real-world setting.

**Design:**

This interim analysis reports outcomes at enrollment in the prospective, observational Tumor-Induced Osteomalacia Disease Monitoring Program (NCT04783428), stratified by burosumab treatment status at enrollment.

**Setting:**

Tertiary care centers in the United States and Argentina.

**Patients:**

Patients with TIO from the Disease Monitoring Program.

**Exposure:**

Burosumab treatment.

**Main outcome measure(s):**

Biochemistry measurements and patient-reported health-related quality of life (pain, fatigue, and physical function).

**Results:**

21 patients with TIO (61.9% female, median [Q1, Q3] age 49.7 years [41.6, 57.3]) were included. At enrollment, 11 (52.4%) patients were treated with burosumab, for a median (Q1, Q3) duration of 4.1 years (0.8, 7.0). Burosumab-treated patients had significantly higher serum phosphate and 1,25-dihydroxyvitamin D levels, and nonsignificantly lower alkaline phosphatase levels, compared with patients not treated with burosumab. Patients treated with burosumab reported significantly lower median Brief Pain Inventory scores, nonsignificantly lower Brief Fatigue Inventory scores, and nonsignificantly higher Patient-Reported Outcomes Measurement Information System Physical Function and Short-Form-36 scores, indicating lower symptom severity and better health-related quality of life.

**Conclusion:**

This study suggests favorable biochemical and patient-reported outcomes in patients with TIO treated with burosumab.

Tumor-induced osteomalacia (TIO) is an ultra-rare condition caused by tumors, typically phosphaturic mesenchymal tumors (PMT), secreting fibroblast growth factor-23 (FGF23) (1-3). Elevated FGF23 levels lead to impaired renal phosphate reabsorption resulting in chronic hypophosphatemia and low serum 1,25-dihydroxyvitamin D levels (4, 5). The clinical manifestations of TIO include severe musculoskeletal morbidities such as fractures and proximal muscle weakness, pain, fatigue, and impaired mobility (6).

Patients with TIO often experience lengthy diagnostic delays, due to limited disease awareness among providers because of the rarity of the condition and symptom overlap with other musculoskeletal, rheumatologic, or neurologic disorders (6-8). Diagnosis often involves visits to multiple specialists, a range of biochemical tests (including serum phosphate, intact FGF23, and tubular maximum phosphate/glomerular filtration rate), and structural or functional imaging (including magnetic resonance imaging, octreoscans, DOTATATE-/FDG-positron emission tomography/computed tomography) (9, 10).

Where possible, complete tumor resection is the definitive treatment for TIO. Alternatively, image guided ablation may be used to treat inoperable or residual tumors. However, localizing the causative tumor(s) is often difficult due to small size, variable tumor location, and limited sensitivity of detection methods. For patients with TIO whose tumors cannot be localized or curatively resected, the traditional treatment has been multiple daily doses of oral phosphate and active vitamin D analogs (3). However, this therapy is associated with tolerability issues and complications, such as gastrointestinal distress, hypercalcemia, nephrocalcinosis, and tertiary hyperparathyroidism (11).

Burosumab, a fully human monoclonal antibody which targets and inhibits FGF23, is indicated for treatment of TIO where tumors cannot be localized or curatively resected (12). It was first approved for treatment of TIO in adult and pediatric patients in June 2020 and is currently the only United States (US) Food and Drug Administration approved pharmaceutical treatment for TIO. Two phase II single-arm clinical trials demonstrated that burosumab restores phosphate homeostasis and leads to improvements in fracture healing, functional mobility, pain, and health-related quality of life in patients with TIO (12, 13).

Because TIO is ultra-rare, there are limited data describing its natural history and disease progression. The TIO Disease Monitoring Program (DMP) was developed to characterize the TIO disease phenotype, including its presentation and course, prospectively over a 10-year period. The current study utilizes real-world data from the TIO DMP baseline visit to explore the differences in outcomes in patients with TIO treated with burosumab vs those not treated with burosumab and to characterize the burden of disease.

## Materials and methods

### Data source

The TIO DMP (NCT04783428) is a 10-year prospective, multicenter, observational study of patients with TIO from the US and Argentina, initiated on 31 January 2022. It was established to monitor the clinical course of the disease and to assess progression over time irrespective of treatment status, as well as to assess the long-term safety and effectiveness of burosumab. Demographic, biochemical, clinical, disease severity, patient-reported outcomes (PROs), treatment, and disease progression data are collected (14). The study population comprises patients with TIO, who may or may not be receiving treatment with burosumab or other treatment options at or prior to study enrollment or at any point in the study. To be included, patients must have had a clinical diagnosis of TIO based on the presence of an underlying PMT and/or confirmed by at least 2 biochemical disease parameters: tubular maximum phosphate/glomerular filtration rate <2.5 mg/dL, fasting serum phosphate level <2.5 mg/dL, intact FGF23 level ≥100 pg/mL, or C-terminal FGF23 ≥ 200 RU/mL. Additionally, for patients in whom the causative PMT had never been localized, documented evidence of negative genetic testing for other hereditary hypophosphatemic disorders was necessary. Patients in the TIO DMP may receive treatment with burosumab through routine clinical prescription but treatment was not required nor provided as part of the DMP.

### Data collection

This study was an interim analysis of data obtained from the TIO DMP, including demographics, medical history, biochemistry, and PROs (Patient-Reported Outcomes Measurement Information System (PROMIS) Physical Function (15), Brief Pain Inventory (BPI) (16), Brief Fatigue Inventory (BFI) (17), and Short Form-36 version 2 (SF-36v2) (18)).

The protocol was approved at each site by local institutional ethical review boards. Informed consent was provided by all participating patients prior to enrollment.

Blood and urine samples were collected after a minimum 4-hour fast and prior to drug administration (if applicable). Blood (serum phosphate, alkaline phosphatase, creatinine, calcium, 1,25-dihydroxyvitamin D, and parathyroid hormone) and urine (calcium and creatinine excretion) tests were performed at a central laboratory in the US and locally in Argentina.

For burosumab, the prescribed dose (mg) was recorded at the date of administration closest prior to enrollment in the TIO DMP.

### Analyses

Analyses were conducted with Stata version 18/18.5/19.5 (StataCorp, College Station, TX). All programming code was developed and logged to ensure replicability, as well as reviewed by an independent programmer for quality control.

Mean, standard deviation (SD), median, first and third quartiles (Q1, Q3), minimum, and maximum, are reported for continuous variables. Number and percentage of patients are reported for categorical variables. Mann–Whitney U tests were used to compare continuous biochemistry and PRO variables by burosumab status. All statistical tests were 2-sided in nature, and a *P*-value of ≤.05 was considered statistically significant. Serum phosphate results were categorized as high, normal, or low based on country-specific ranges (US: 2.2 mg/dL ≤ Normal ≤ 5.1 mg/dL, Argentina: 2.5 mg/dL ≤ Normal ≤4.5 mg/dL) (19). The number and percentage of patients below, within, and above the normal range for serum phosphate were assessed. PRO scores were derived as per scoring guidelines (PROMIS Physical Function (15), BPI (16), BFI (17), and SF-36 v2 (18)).

## Results

### Demographics

A total of 21 patients with confirmed TIO were enrolled, of which 13 (61.9%) were female, with a median (Q1, Q3) age of 49.7 (41.6, 57.3) years (Table 1). There was a roughly even split across countries: 11 US, 10 Argentina.

**Table 1:** Demographic characteristics

	Overall (N = 21)	Treated with burosumab at DMP enrollment (N = 11)	Not treated with burosumab at DMP enrollment (N = 10)
Age at enrollment (years)			
N	21	11	10
Mean (SD)	49.7 (13.5)	55.0 (15.1)	43.8 (8.8)
Median (Q1, Q3)	49.7 (41.6, 57.3)	57.3 (46.6, 67.9)	46.3 (38.9, 49.7)
Minimum, maximum	19.3, 69.7	19.3, 69.7	27.9, 54.4
Sex, n (%)			
N	21	11	10
Female	13 (61.9)	6 (54.5)	7 (70.0)
Male	8 (38.1)	5 (45.5)	3 (30.0)
Country, n (%)			
N	21	11	10
US	11 (52.4)	7 (63.6)	4 (40.0)
Argentina	10 (47.6)	4 (36.4)	6 (60.0)
Race, n (%)			
N	21	11	10
White	21 (100.0)	11 (100.0)	10 (100.0)
Ethnicity, n (%)			
N	21	11	10
Not Hispanic or Latin	11 (52.4)	7 (63.6)	4 (40.0)
Hispanic or Latin	10 (47.6)	4 (36.4)	6 (60.0)
Body mass index (kg/m^2^)			
N	21	11	10
Mean (SD)	32.7 (9.1)	32.1 (7.7)	33.3 (10.9)
Median (Q1, Q3)	30.3 (26.5, 38.7)	33.4 (25.9, 38.7)	28.6 (26.5, 40.5)
Minimum, maximum	17.9, 57.1	17.9, 42.0	20.5, 57.1

Abbreviations: DMP, Disease Monitoring Program; kg, kilogram, m, meter; Q, quartile; SD, standard deviation; US, United States.

Overall, 11 (52.4%) patients were actively being treated with burosumab at enrollment. Of the 10 patients not treated with burosumab, one had previously been treated with burosumab but had discontinued prior to DMP enrollment (further details in Table 2). Demographic characteristics, except for age, were similar for patients treated and not treated with burosumab; burosumab-treated patients were older, with a median age (Q1, Q3) of 57.3 (46.6, 67.9) years compared with 46.3 (38.9, 49.7) years for patients not treated. The results stratified by use of oral phosphate and/or active vitamin D analogs at DMP enrollment are presented in the supplementary materials (20).

**Table 2:** Medication use

	Overall (N = 21)	Treated with burosumab at DMP enrollment (N = 11)	Not treated with burosumab at DMP enrollment (N = 10)
Burosumab use prior to enrollment into the DMP, n (%)			
N	21	11	10
Yes	12 (57.1)	11 (100.0)	1 (10.0)*^a^*
No	9 (42.9)	0 (0.0)	9 (42.9)
Burosumab discontinuation prior to DMP enrollment of those prescribed burosumab ever, n (%)			
N	12	11	1
No discontinuation	8 (66.7)	8 (72.7)	0 (0.0)
Temporary discontinuation	3 (25.0)	3 (27.3)	0 (0.0)
Full discontinuation	1 (8.3)	0 (0.0)	1 (100.0)
Time since earliest burosumab use (years) of those treated with burosumab at DMP enrollment			
N	11	11	—
Mean (SD)	4.0 (2.9)	4.0 (2.9)	—
Median (Q1, Q3)	4.1 (0.8, 7.0)	4.1 (0.8, 7.0)	—
Minimum, maximum	0.0, 7.3	0.0, 7.3	—
Burosumab dose (mg) nearest to DMP enrollment			
N	9	9	—
Mean (SD)	53.3 (22.4)	53.3 (22.4)	—
Median (Q1, Q3)	60.0 (30.0, 60.0)	60.0 (30.0, 60.0)	—
Minimum, maximum	30.0, 90.0	30.0, 90.0	—
Treatment with oral phosphate ever, n (%)			
N	21	11	10
Yes	17 (81.0)	10 (90.9)	7 (70.0)
No	4 (19.0)	1 (9.1)	4 (19.0)
Discontinued oral phosphate (of those ever treated with oral phosphate), n (%)			
N	17	10	7
Yes	14 (82.4)	10 (100.0)	4 (57.1)
No	3 (17.6)	0 (0.0)	3 (42.9)
Reason for discontinuing oral phosphate, n (%)			
N	14	10	4
Stopped phosphate to start burosumab	8 (57.1)	8 (80.0)	0 (0.0)
Side effects or complications	2 (14.3)	1 (10.0)	1 (25.0)
Expense	1 (7.1)	1 (10.0)	0 (0.0)
Challenge after PMT resection	1 (7.1)	0 (0.0)	1 (25.0)
Temporary discontinuation while lab result was processed	1 (7.1)	0 (0.0)	1 (25.0)
Surgery	1 (7.1)	0 (0.0)	1 (25.0)
Unknown	1 (7.1)	1 (10.0)	0 (0.0)
Treatment with active vitamin D ever, n (%)			
N	21	11	10
Yes	18 (85.7)	10 (90.9)	8 (80.0)
No	3 (14.3)	1 (9.1)	2 (20.0)
Discontinued active vitamin D (of those receiving active vitamin D), n (%)			
N	18	10	8
Yes	12 (66.7)	10 (100.0)	2 (25.0)
No	6 (33.3)	0 (0.0)	6 (75.0)
Reason for discontinuing active vitamin D, n (%)			
N	12	10	2
Stopped active vitamin D to start burosumab	8 (66.7)	8 (80.0)	0 (0.0)
Expensive	1 (8.3)	1 (10.0)	0 (0.0)
Side effects or complications	1 (8.3)	1 (10.0)	0 (0.0)
Surgery	1 (8.3)	0 (0.0)	1 (50.0)
Considered in remission	1 (8.3)	0 (0.0)	1 (50.0)
Unknown	1 (8.3)	1 (10.0)	0 (0.0)
Pain medication at enrollment, n (%)			
N	21	11	10
Yes	10 (47.6)	4 (36.4)	6 (60.0)
No	11 (52.4)	7 (63.6)	4 (40.0)
Type of pain medication at enrollment, n (%)			
N	21	11	10
Nonsteroidal anti-inflammatory drugs	9 (42.9)	3 (27.3)	6 (60.0)
Steroid	3 (14.3)	0 (0.0)	3 (30.0)
Acetaminophen/paracetamol	2 (9.5)	0 (0.0)	2 (20.0)
Opioids	2 (9.5)	1 (9.1)	1 (10.0)
Neuropathic pain medications/antidepressants	0 (0.0)	0 (0.0)	0 (0.0)

Abbreviations: DMP, Disease Monitoring Program; mg, milligram; Q, quartile; SD, standard deviation.

^*a*^Patient was treated with burosumab for 3 months and discontinued burosumab 19 months prior to DMP enrollment.

### Treatment patterns

Patients who were treated with burosumab had a median (Q1, Q3) treatment duration of 4.1 (0.8, 7.0) years, with a median (Q1, Q3) dose of 60.0 mg (30.0, 60.0) (Table 2). Most patients treated with burosumab had previously been treated with active vitamin D and oral phosphate supplements (10 [90.9%]). Of those not treated with burosumab at enrollment, 9 (90.0%) patients had received active vitamin D and/or oral phosphate in the past, and one had previously been treated with burosumab (discontinued 19 months prior to DMP enrollment). Pain medication use was reported at enrollment by 4 patients (36.4%) treated with burosumab and 6 patients (60.0%) not treated with burosumab.

### Biochemistry

**Figure 1:**
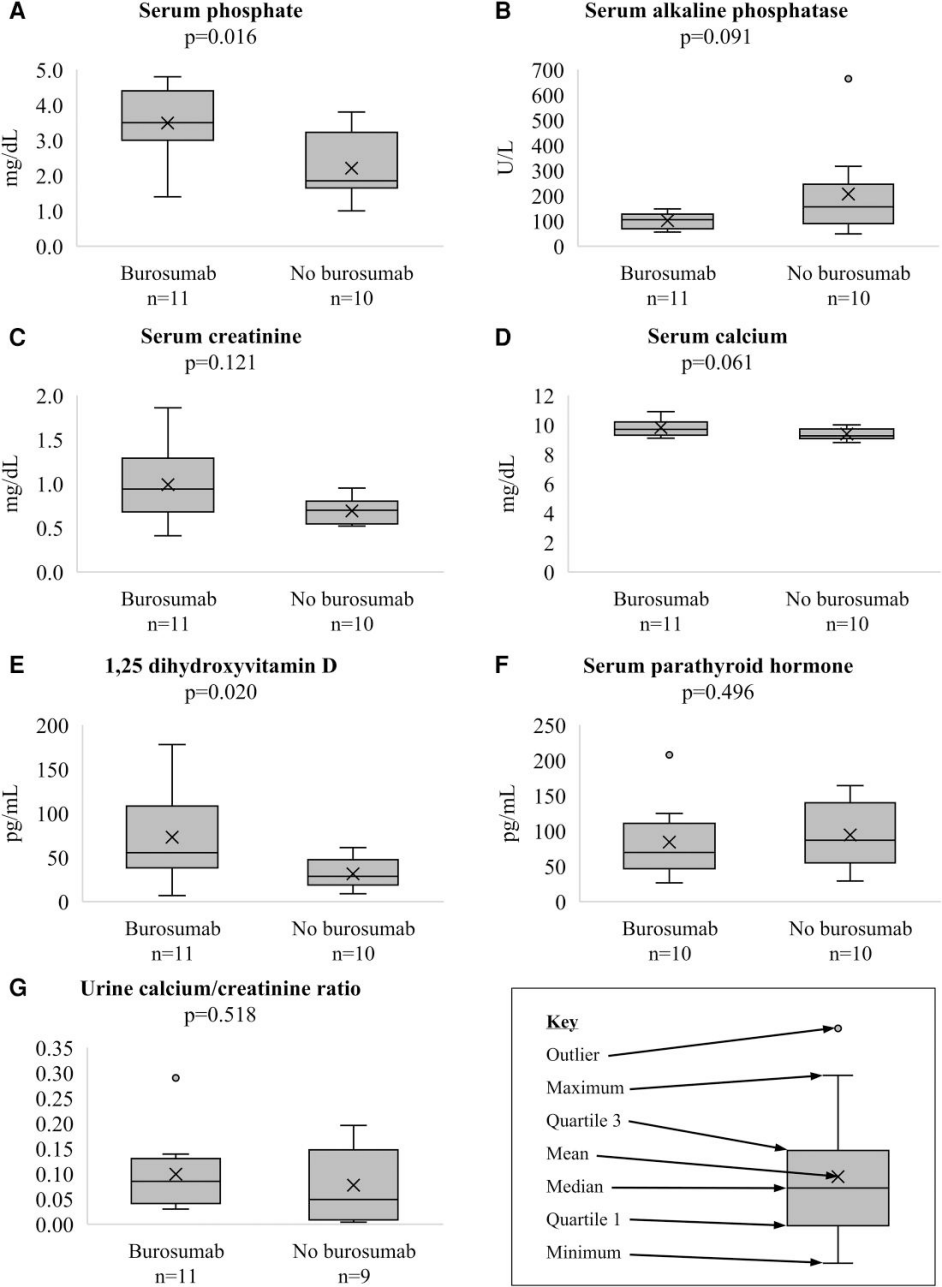
Biochemistry test results for patients treated and not treated with burosumab (A) serum phosphate, (B) serum alkaline phosphatase, (C) serum creatinine, (D) serum calcium, (E) 1,25 dihydroxyvitamin D, (F) serum parathyroid hormone, and (G) urine calcium/creatinine ratio. dL, deciliter; L, liter; mg, milligram; mL, milliliter; pg, picogram; U, [international] unit.

Median (Q1, Q3) serum phosphate concentration was significantly higher in patients treated with burosumab (3.5 [1.0, 3.5] vs no burosumab 2.2 [1.0, 1.9], *P* = .016) (Fig. 1)—within the normal range in 9 (81.8%) patients treated compared with 3 (30.0%) patients who were not treated with burosumab. Additionally, the median 1,25-dihydroxyvitamin D level was significantly higher in patients treated (55.4 [38.3, 108.3]) vs those not treated with burosumab (28.6 [21.1, 47.1], *P* = .020) (Fig. 1). Serum alkaline phosphatase levels were nonsignificantly lower in patients treated vs those not treated with burosumab (*P* > .05). Serum creatinine, parathyroid hormone, and urinary calcium/creatinine ratio values were not significantly different between the groups (Fig. 1).

### Patient-reported outcomes

**Figure 2:**
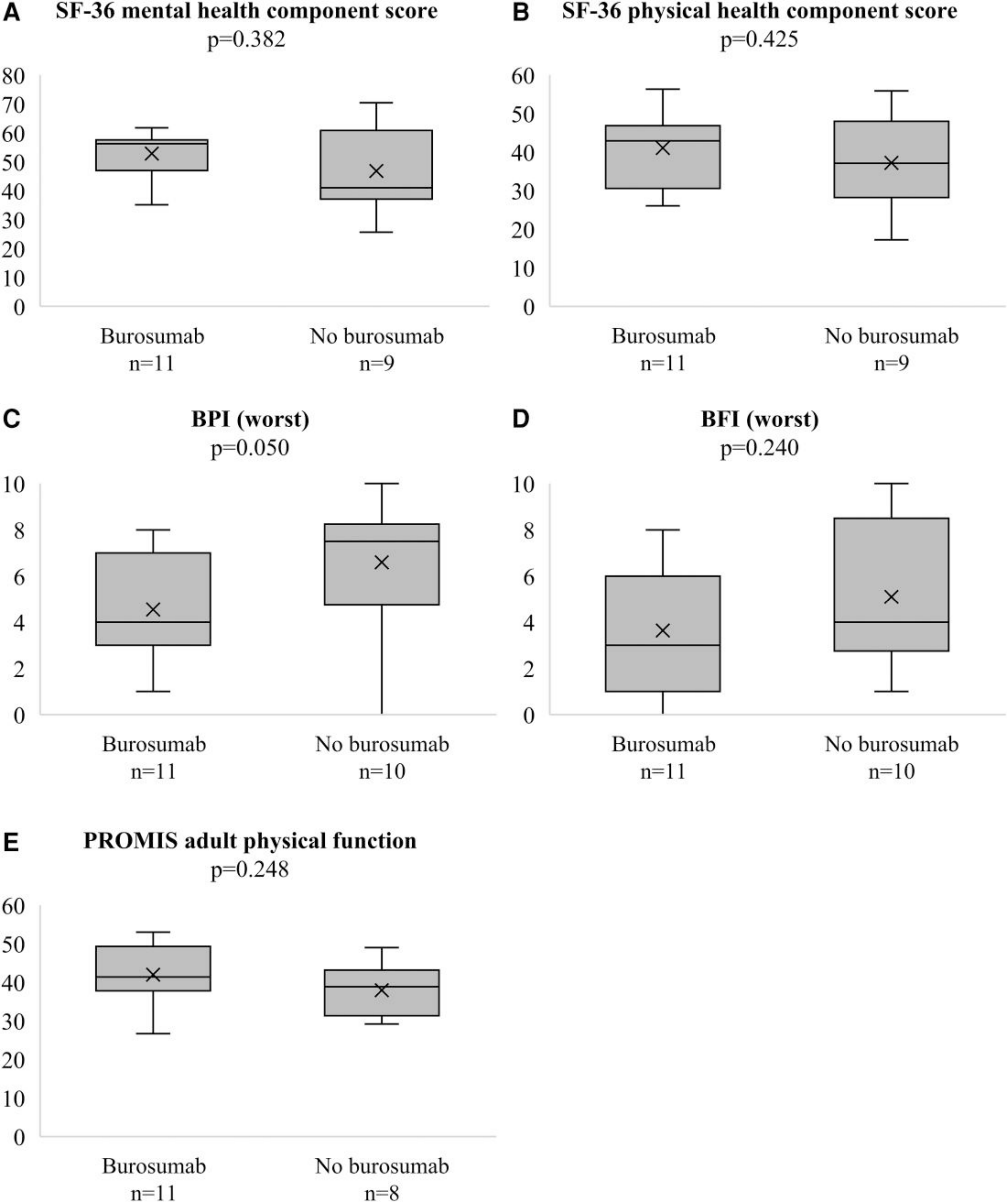
PROs for patients treated and not treated with burosumab (A) SF-36 mental health component score, (B) SF-36 physical health component score, (C) BPI (worst), (D) BFI (worst), and (E) PROMIS adult physical function. BFI, Brief Fatigue Inventory; BPI, Brief Pain Inventory; PRO, patient-reported outcome; PROMIS, Patient-Reported Outcomes Measurement Information System; SF-36, short form-36.

**Figure 3:**
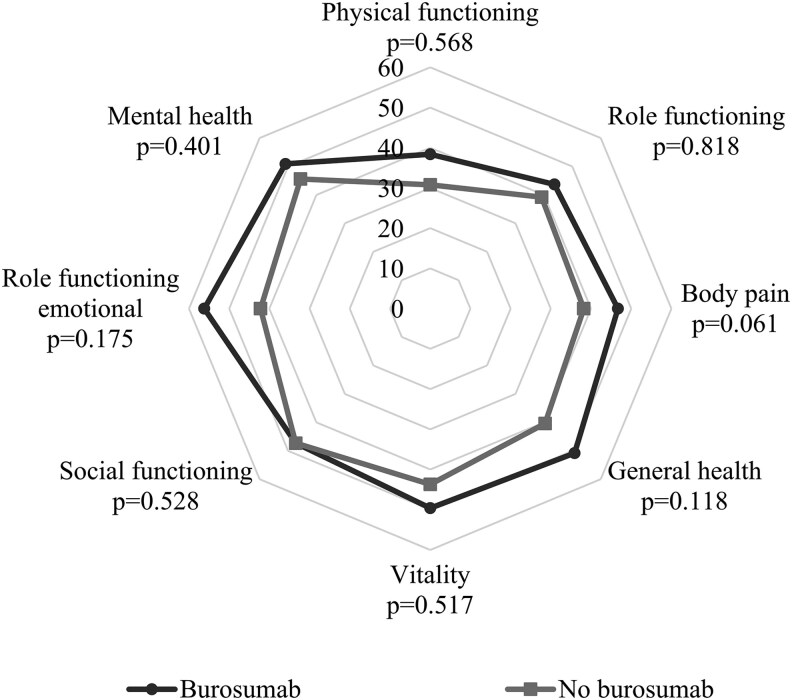
Median SF-36 domain scores. Higher scores are indicative of better health. SF-36, short form-36.

Patients treated with burosumab at enrollment had significantly lower pain scores (BPI worst pain) compared with patients not treated with burosumab (median [Q1, Q3]: burosumab 4.0 [3.0, 7.0], no burosumab 7.5 [5.0, 8.0], *P* = .050) (Fig. 2). BFI worst fatigue (burosumab 3.0 [1.0, 6.0], no burosumab 4.0 [3.0, 8.0], *P* = .240) and PROMIS adult physical function (burosumab 41.4 [37.8, 49.3], no burosumab 38.8 [31.5, 42.5], *P* = .248) scores suggested less fatigue and better physical function in those treated with burosumab, respectively. SF-36 mental health component (burosumab 56.2 [47.0, 57.6], no burosumab 41.0 [37.6, 59.9], *P* = .382) and physical health component (burosumab 42.9 [30.6, 46.8], no burosumab 37.2 [28.4, 44.0], *P* = .425) scores favored those treated with burosumab (Fig. 2). Likewise across most SF-36 domains, patients treated with burosumab had a favorable median score compared with patients not treated with burosumab (*P* > .05), with the exception of the social functioning domain (*P* = .528) (Fig. 3). Hence, overall for PROs, there was a trend toward more favorable scores in those treated with burosumab, reaching statistical significance for BPI worst pain.

### TIO disease and clinical manifestations

Sixteen (76.2%) patients had a tumor location identified, and 3 had more than 1 tumor (Table 3). The most frequently reported locations for tumors were the lower limb (6 [37.5%]), head/neck (5 [31.3%]), and trunk (5 [31.3%]) regions, and the distribution of tumors was similar between groups. Among patients with a tumor location identified, the majority (12 [75.0%]) had undergone at least 1 tumor resection. The median (Q1, Q3) number of resections was 3 (2.0, 3.0) for patients treated with burosumab and one (1.0, 1.0) for those not treated with burosumab of resections.

**Table 3:** Tumor characteristics and surgical history

	Overall (N = 21)	Treated with burosumab at DMP enrollment (N = 11)	Not treated with burosumab at DMP enrollment (N = 10)
Patients with tumor location identified, n (%)			
N	21	11	10
Yes	16 (76.2)	8 (72.7)	8 (80.0)
No	5 (23.8)	3 (27.3)	2 (20.0)
Number of tumors in those with tumor location identified, n (%)			
N	16	8	8
1	13 (81.3)	5 (62.5)	8 (100.0)
2	1 (6.3)	1 (12.5)	0 (0.0)
3	2 (12.5)	2 (25.0)	0 (0.0)
Tumor location in those with a tumor location identified, n (%)			
N	16	8	8
Lower limb	6 (37.5)	3 (37.5)	3 (37.5)
Head/neck	5 (31.3)	3 (37.5)	2 (25.0)
Trunk	5 (31.3)	3 (37.5)	2 (25.0)
Hip/pelvis	2 (12.5)	2 (25.0)	0 (0.0)
Upper limb	2 (12.5)	1 (12.5)	1 (12.5)
Patients with at least 1 tumor resection of patients with a tumor location identified, n (%)			
N	16	8	8
Yes	12 (75.0)	6 (75.0)	6 (75.0)
No	4 (25.0)	2 (25.0)	2 (25.0)
Number of tumor resections among patients with a localized and resected tumor			
N	12	6	6
Mean (SD)	2.0 (1.3)	2.8 (1.3)	1.2 (0.4)
Median (Q1, Q3)	1.5 (1.0, 3.0)	3.0 (2.0, 3.0)	1.0 (1.0, 1.0)
Minimum, maximum	1.0, 5.0	1.0, 5.0	1.0, 2.0
History of radiotherapy, n (%)			
N	21	11	10
Yes	1 (4.8)	1 (9.1)	0 (0.0)
No	20 (95.2)	10 (90.9)	10 (100.0)

Abbreviations: DMP, Disease Monitoring Program; Q, quartile; SD, standard deviation.

Prior to TIO diagnosis, patients were most commonly managed by an orthopedist (13 [61.9%]), endocrinologist (9 [42.9%]), and/or rheumatologist (9 [42.9%]) (20). However, at the time of enrollment, for most patients (20 [95.2%]) an endocrinologist managed their TIO-related symptoms. Just under half of patients (9 [42.9%]) reported ambulatory and assistive device use.

Overall, a high percentage of patients had bone pain (20 [95.2%]), muscle weakness (16 [76.2%]), fatigue (13 [61.9%]), and/or difficulty ambulating (15 [71.4%]) as presenting clinical features (Table 4). Over half had a history of fracture (16 [76.2%]). A history of kidney stones was reported for four (19%) patients. Overall, clinical history and symptoms were comparable between groups.

**Table 4:** Clinical history and presenting symptoms at diagnosis

	Overall (N = 21)	Treated with burosumab at DMP enrollment (N = 11)	Not treated with burosumab at DMP enrollment (N = 10)
Clinical symptoms, n (%)			
N	21	11	10
Bone pain	20 (95.2)	11 (100.0)	9 (90.0)
Muscle weakness	16 (76.2)	7 (63.6)	9 (90.0)
Difficulty ambulating	15 (71.4)	5 (45.5)	10 (100.0)
Fatigue	13 (61.9)	5 (45.5)	8 (80.0)
Fracture(s)	13 (61.9)	8 (72.7)	5 (50.0)
Short stature (poor growth)	2 (9.5)	2 (18.2)	0 (0.0)
Height loss	1 (4.8)	1 (9.1)	0 (0.0)
TIO-specific skeletal history, n (%)			
N	21	11	10
Osteoarthritis	5 (23.8)	4 (36.4)	1 (10.0)
Enthesopathy/bone spurs/osteophytes	4 (19.0)	1 (9.1)	3 (30.0)
Spinal cord compression	1 (4.8)	0 (0.0)	1 (10.0)
Spinal stenosis	1 (4.8)	1 (9.1)	0 (0.0)
Skeletal surgical history, n (%)			
N	12	6	6
Fracture surgery repair	10 (83.3)	5 (83.3)	5 (83.3)
Joint replacement	3 (25.0)	3 (50.0)	0 (0.0)
Bone shaving/bone spur removal	2 (16.7)	0 (0.0)	2 (33.3)
Spinal fusion	3 (25.0)	2 (33.3)	1 (16.7)
Fracture location, n (%)			
N	21	11	10
Hip/pelvis	8 (38.1)	5 (45.5)	3 (30.0)
Ribs	7 (33.3)	4 (36.4)	3 (30.0)
Femur	6 (28.6)	3 (27.3)	3 (30.0)
Foot	6 (28.6)	2 (18.2)	4 (40.0)
Vertebrae	5 (23.8)	3 (27.3)	2 (20.0)
Tibia	3 (14.3)	1 (9.1)	2 (20.0)
Ankle	1 (4.8)	0 (0.0)	1 (10.0)
Fibula	1 (4.8)	1 (9.1)	0 (0.0)
Wrist	1 (4.8)	1 (9.1)	0 (0.0)
Hand	1 (4.8)	0 (0.0)	1 (10.0)
Humerus	1 (4.8)	1 (9.1)	0 (0.0)
Renal history, n (%)			
N	21	11	10
Kidney stone history	4 (19.0)	2 (18.2)	2 (20.0)
Hyperparathyroidism history	3 (14.3)	2 (18.2)	1 (10.0)
Chronic kidney disease history	1 (4.8)	1 (9.1)	0 (0.0)
Hypercalcemia history	1 (4.8)	1 (9.1)	0 (0.0)
Nephrocalcinosis history	0 (0.0)	0 (0.0)	0 (0.0)
Renal insufficiency history	0 (0.0)	0 (0.0)	0 (0.0)

Abbreviations: DMP, Disease Monitoring Program; TIO, tumor-induced osteomalacia; Q, quartile; SD, standard deviation.

### Diagnosis

The median (Q1, Q3) time from TIO diagnosis to enrollment for the whole study cohort was 3.6 (1.1, 8.6) years (Table 5). This interval was longer for patients receiving burosumab (8.6 [2.7, 17.2] years) compared with patients not receiving burosumab (1.1 [0.2, 4.2] years) at enrollment. Diagnoses based on biochemical evidence (such as elevated FGF23 levels or hypophosphatemia) and/or imaging/tumor location were common—18 (85.7%) and 15 (71.4%) patients, respectively. Most patients (18 [85.7%]) had at least one prior misdiagnosis, including osteoporosis (6 [28.6%]), spondyloarthritis (4 [19.0%]), and cancer (4 [19.0%]).

**Table 5:** Diagnosis of TIO

	Overall (N = 21)	Treated with burosumab at DMP enrollment (N = 11)	Not treated with burosumab at DMP enrollment (N = 10)
Time since TIO diagnosis to DMP enrollment (years)			
N	21	11	10
Mean (SD)	6.6 (7.7)	10.7 (8.8)	2.1 (2.1)
Median (Q1, Q3)	3.6 (1.1, 8.6)	8.6 (2.7, 17.2)	1.1 (0.2, 4.2)
Minimum, maximum	(0.2, 28.8)	(0.4, 28.8)	(0.2, 5.6)
Method of diagnosis confirmation, n (%)			
N	21	11	10
Biochemical evidence, such as hypophosphatemia, elevated FGF23	18 (85.7)	9 (81.8)	9 (90.0)
Location of tumor through imaging	15 (71.4)	9 (81.8)	6 (60.0)
Biopsy	1 (4.8)	1 (9.1)	0 (0.0)
Location of tumor through surgery	1 (4.8)	0 (0.0)	1 (10.0)
Prior misdiagnosis, n (%)			
N	21	11	10
Yes	18 (85.7)	10 (90.9)	8 (80.0)
No	3 (14.3)	1 (9.1)	2 (20.0)
Prior misdiagnosis, n (%)			
N	21	11	10
Osteoporosis	6 (28.6)	2 (18.2)	4 (40.0)
Spondyloarthritis	4 (19.0)	3 (27.3)	1 (10.0)
Cancer	4 (19.0)	2 (18.2)	2 (20.0)
Neurological/pain syndrome	3 (14.3)	1 (9.1)	2 (20.0)
Fibromyalgia	2 (9.5)	2 (18.2)	0 (0.0)
Osteoarthritis	2 (9.5)	1 (9.1)	1 (10.0)
Hyperparathyroidism	1 (4.8)	1 (9.1)	0 (0.0)
Other spine/joint condition	1 (4.8)	1 (9.1)	0 (0.0)
Metabolic disorder	1 (4.8)	1 (9.1)	0 (0.0)
Parasitic disease	1 (4.8)	1 (9.1)	0 (0.0)

Abbreviations: DMP, Disease Monitoring Program; FGF23, fibroblast growth factor-23; TIO, tumor-induced osteomalacia; Q, quartile; SD, standard deviation.

### Productivity

At DMP enrollment, 9 (42.9%) patients were in full-time employment, and 6 (28.6%) patients were not in current employment or studying (Table 6). Of those in employment with available data, 1 patient missed 180 workdays in the last 6 months due to feeling unwell, and there was an overall median (Q1, Q3) of 0.0 (0.0, 30.0) and mean (SD) of 27.3 (56.2) of missed workdays. Additionally, four (33.3%) patients who were in employment had a reduced responsibility level at work.

**Table 6:** Productivity

	Overall (N = 21)	Treated with burosumab at DMP enrollment (N = 11)	Not treated with burosumab at DMP enrollment (N = 10)
Employment, n (%)			
N	21	11	10
Full-time employment	9 (42.9)	4 (36.4)	5 (50.0)
Part-time employment	3 (14.3)	1 (9.1)	2 (20.0)
Full-time university or professional studies	1 (4.8)	1 (9.1)	0 (0.0)
Part-time university or professional studies	2 (9.5)	0 (0.0)	2 (20.0)
Not currently employed or studying	6 (28.6)	5 (45.5)	1 (10.0)
Workdays missed due to not feeling well in the last 6 months, among those in employment at enrollment*^a^*			
N	11	5	6
Mean (SD)	27.3 (56.2)	6.0 (13.4)	45.0 (73.1)
Median (Q1, Q3)	0.0 (0.0, 30.0)	0.0 (0.0, 0.0)	5.0 (0.0, 80.0)
Minimum, maximum	(0.0, 180.0)	(0.0, 30.0)	(0.0, 180.0)
Reduced responsibility level at work, n (%)			
N	12	5	7
Yes	4 (33.3)	1 (20.0)	3 (42.9)
No	8 (66.7)	4 (80.0)	4 (57.1)

Abbreviations: DMP, Disease Monitoring Program; Q, quartile; SD, standard deviation.

^*a*^n = 1 missing; in employment but did not answer how many workdays had been missed.

## Discussion

This study offers the first direct comparison of outcomes between patients treated vs not treated with burosumab, building on previous single-arm phase II studies and describing the burden of TIO using real-world data. Overall, results suggest favorable biochemical and patient-reported outcomes in patients treated with burosumab, despite being older with longer disease duration. Importantly, these results also highlight the substantial burden of TIO, including impaired quality of life, persistent symptoms, and a challenging diagnostic odyssey.

At TIO DMP enrollment, patients treated with burosumab had significantly higher serum phosphate and 1,25-dihydroxyvitamin D levels compared with those not treated with burosumab. Further, a trend toward lower serum alkaline phosphatase values in the burosumab-treated group was also evident. Additionally, symptom profiles including pain, fatigue, physical functioning, and HRQoL were consistently lower in those treated with burosumab, although differences were not statistically significant in most cases. The most striking difference was seen for pain, favoring patients treated with burosumab across both BPI and SF-36 body pain domain measures. These results support findings from clinical trials, which showed normalization of serum phosphate levels and improvement in patient-reported symptoms following burosumab treatment (12, 13, 21).

Patients enrolled in the TIO DMP showed lower physical function (PROMIS-PF) and SF-36 physical health component scores compared with the US general population (mean of 50 and threshold of 47 for impaired HRQoL, respectively) (22-24). A high percentage of patients in this study experienced symptoms of bone pain, muscle weakness, fatigue, and/or fractures, and difficulty ambulating, in line with previous studies (8, 25). Additionally, just under half of patients (42.9%) were using ambulatory and assistive devices, which highlight the impaired physical function and productivity that many patients with TIO experience. Employment status at DMP enrollment showed that fewer than half of patients were in full-time work, while work productivity among employed patients was frequently affected, including missed workdays and reduced responsibility levels.

The age and clinical symptoms of patients in this study were similar to those reported in published systematic reviews of TIO; however, the DMP included a higher percentage of females (7, 8, 25). Among those with at least 1 tumor location specified, most had undergone at least 1 tumor resection, which highlights potential challenges for curative resection of certain tumors. Many patients in the TIO DMP had consulted multiple clinicians prior to being diagnosed with TIO, reflecting the diagnostic challenges associated with the disease and its symptom overlap with other rheumatological and musculoskeletal disorders, as noted in prior studies (6).

This study is the first to compare outcomes in patients with TIO treated vs not treated with burosumab, and, unlike previous real-world studies (7), provides a thorough profile of patient-related outcomes. The study population was small (N = 21), reflecting the rarity of TIO, resulting in a limited statistical power for our comparisons. Other limitations include the cross-sectional nature of the study and potential confounding. Patients treated with burosumab initiated treatment prior to enrollment and therefore treatment duration varied; no minimum treatment duration was required for the burosumab cohort. Productivity outcomes were self-reported by patients; their accuracy depends on reliable self-reporting. The comparisons in this analysis were not adjusted for other factors; patients treated with burosumab at DMP enrollment were older, had a longer time since diagnosis compared with patients not treated with burosumab, and burosumab treatment duration was not uniform. However, based on these characteristics, the favorable biochemistry and PROs in this group strengthen the conclusions of the study. Future research will investigate outcomes with longitudinal data from the TIO DMP, whereby the impact of burosumab on clinically relevant outcomes can be further understood.

## Conclusions

This interim analysis of the TIO DMP suggests favorable biochemical and patient-reported outcomes in patients with TIO treated vs not treated with burosumab. Findings from this study also underscore the high burden of disease in patients with TIO.

## Data Availability

Some or all datasets generated during and/or analyses during the current study are not publicly available but are available from the corresponding author on reasonable request.
